# Root Bacteria Recruited by *Phragmites australis* in Constructed Wetlands Have the Potential to Enhance Azo-Dye Phytodepuration

**DOI:** 10.3390/microorganisms7100384

**Published:** 2019-09-24

**Authors:** Valentina Riva, Francesca Mapelli, Evdokia Syranidou, Elena Crotti, Redouane Choukrallah, Nicolas Kalogerakis, Sara Borin

**Affiliations:** 1 Department of Food, Environmental and Nutritional Sciences, Università degli Studi di Milano, 20133 Milano, Italy; valentina.riva@unimi.it (V.R.); francesca.mapelli@unimi.it (F.M.); elena.crotti@unimi.it (E.C.); 2 School of Environmental Engineering, Technical University of Crete, Polytecneioupolis, 73100 Chania, Greece; evdokiasyranidou@gmail.com (E.S.); nicolas.kalogerakis@enveng.tuc.gr (N.K.); 3 Hassan II, Salinity and Plant Nutrition Laboratory, Institut Agronomique et Vétérinaire, 86150 Agadir, Morocco; redouane53@yahoo.fr

**Keywords:** plant growth promoting bacteria, micropollutants, *Juncus acutus*, reactive black-5, azo-dye decolorization, wastewater treatment

## Abstract

The microbiome associated with plants used in phytodepuration systems can boost plant growth and services, especially in ecosystems dealing with recalcitrant compounds, hardly removed via traditional wastewater (WW) treatments, such as azo-dyes used in textile industry. In this context, we aimed to study the cultivable microbiome selected by *Phragmites australis* plants in a Constructed Wetland (CW) in Morocco, in order to obtain candidate inoculants for the phytodepuration of azo-dye contaminated WW. A collection of 152 rhizospheric and endophytic bacteria was established. The strains were phylogenetically identified and characterized for traits of interest in the phytodepuration context. All strains showed Plant Growth Promotion potential in vitro and 67% of them significantly improved the growth of a model plant in vivo compared to the non bacterized control plants. Moreover, most of the isolates were able to grow in presence of several model micropollutants typically found in WW, indicating their potential use in phytodepuration of a wide spectrum of effluents. The six most promising strains of the collection were tested in CW microcosms alone or as consortium: the consortium and two single inocula demonstrated to significantly increase the removal of the model azo-dye Reactive Black 5 compared to the non bacterized controls.

## 1. Introduction

The textile industry is one of the major producers of liquid effluent pollutants, due to the large quantity of water used in its production processes (up to 150 L of water to dye 1 kg of cotton) [1]. In fact, during the dyeing process, a significant fraction of dyes does not bind to the fibers and is released as effluent into the textile wastewaters. Azo-dyes, which are the most used dyes in the textile manufacturing, are xenobiotic compounds highly recalcitrant to degradation processes: their improper discharge in aqueous ecosystems leads to a reduction in water sunlight penetration, which decreases photosynthetic activity and dissolved oxygen concentration, besides having toxic effects on aquatic flora and fauna [2]. These synthetic molecules, which are also applied in other commercial sectors such as printing, cosmetics and food industries [3,4], represent a relevant environmental and health issue, in particular in those countries where wastewater is used for irrigation purposes without prior proper treatments [3]. Reactive azo-dyes are recalcitrant to conventional wastewater treatment processes and in some cases up to 90% of these molecules could remain unprocessed after activated sludge treatment [5]. Moreover, the specific treatment required to implement the removal of azo-dyes in wastewaters [6,7,8] are in most cases expensive and cannot be incorporated in conventional treatment processes [9].

In the Middle East and North Africa (MENA) region, the textile industry represents a relevant sector for the local economy: textile production provides 6% of Gross Domestic Product (GDP) in Egypt and 7% in Morocco and Tunisia [10]. Since this region suffers water scarcity, the use of wastewater treatment plant final effluent for irrigation purposes is a common practice that is expected to increase in parallel to the population and economy growth [10]. However, the quality of treated wastewater is still poor in the MENA countries and many pollutants are insufficiently removed because of the lack of tertiary treatments and the poor plant maintenance: as a result, sewages are often directly discharged into the rivers [11,12]. A promising solution, especially for low-income countries, is represented by Constructed Wetland (CW) systems that exploit biological treatments for the depuration of wastewaters. Compared to the common physicochemical processes used for the removal of azo-dyes from textile wastewater effluents [13], CWs are low cost systems that can be easily operated and maintained because they do not require additional energy and chemicals [14].

CWs are engineered systems that take advantage of purifying processes naturally occurring in wetlands where chemical, physical and biological processes may spontaneously take place at the same time, thanks to the different components of the system and their interactions [15,16,17]. Soil/sediments and plant root apparatus can improve water quality by sorption, sedimentation, phytodegradation and plant uptake of organic and inorganic compounds. Moreover, these matrices provide a large surface area for the growth of complex and diverse microbial communities that play a pivotal role in wetland biological processes [18,19,20].

CWs demonstrated to efficiently decrease the concentration of conventional wastewater components such as organic carbon (chemical and biochemical oxygen demand), total suspended solid (TSS) and nutrients (e.g., ammonia and phosphorus) [14,15]. Recent studies showed that micropollutants, including emerging organic contaminants (EOCs) like pharmaceuticals and personal care products, plasticizers and heavy metals can be removed in CWs [21,22,23]. The biological removal of synthetic dyes was recently showed in floating treatment wetland mesocosms carrying *Phragmites australis* in combination with three dye-degrading bacteria [24]. Furthermore, a *Bacillus* strain, isolated from the rhizosphere of sorghum plants grown in textile wastewater contaminated soil, efficiently decolorized different azo dyes and was indicated as a powerful inoculant for the bioremediation of textile wastewaters [2].

In CW systems, although plants play a significant role in direct uptake of pollutants from wastewaters, the processes of transformation and mineralization of nutrient and organic pollutants greatly depend on the microbial communities associated to their root systems [18]. The root associated microbiome could also counteract the effects on plants (i.e., lower growth and performance) caused by the stressful environmental conditions occurring in phytodepuration systems, mainly due to pollutants’ presence. In this context, the exploitation of Plant Growth Promoting (PGP) bacteria can be a promising strategy for the improvement of CW services. Syranidou and coworkers [25] demonstrated that the bacterial community of *Juncus acutus* plants in a pilot CW study treating bisphenol A (BPA)-contaminated groundwater was enriched in strains with PGP traits that showed tolerance to high concentration of metals and ability to degrade different organic compounds comprising BPA.

The identification and functional characterization of the microorganisms associated to plants commonly used in CW systems is therefore fundamental, not only to predict the degradation potential of the process, but also to find new powerful microbial inoculants that could ameliorate plant services and the overall technology efficiency [22]. In this work, we studied the cultivable fraction of the bacterial community associated to the root system of *Phragmites australis* (common reed), which is widely used for phytodepuration given its ability to grow in different freshwater environments, including the most polluted ones, and to absorb many contaminants thanks to its high lignin and cellulose content [20]. *P. australis* samples collected in a CW plant as a tertiary treatment of municipal WWs were used to establish a collection of bacterial isolates that has been identified and characterized for the ability i) to promote plant growth, ii) to decolorize dyes and iii) to tolerate model micropollutants (heavy metals, bisphenol-A). Basing on the screening results, the most promising strains were selected for a microcosm-scale CW experiment to test their suitability as candidate inoculants for the specific removal of the azo-dye Reactive Black-5 from synthetic textile wastewaters.

## 2. Materials and Methods

### 2.1. Sample Collection

*Phragmites australis* root systems were sampled from triplicate plants growing in a Constructed Wetland plant located in Drarga (Souss-Massa region, southern Morocco) used as municipal wastewater tertiary treatment.

Samples were collected using sterile tools and transferred to the laboratories of the University of Milan within 48 hours. The rhizosphere soil—defined as soil particles tightly adhering to roots (1–3 mm)—was separated from the root tissues in sterile conditions according to the procedure described by Marasco et al. [26].

### 2.2. Bacteria Collection Establishment

The replicated samples (n = 3) of *P. australis* roots and rhizospheric soil were pooled and homogenized prior to endophytic (from surface sterilized roots) and rhizospheric (from rhizosphere) bacteria isolation.

To obtain the endophytic bacterial collection, roots collected from 3 plant specimens (around 1 g/specimen) were vigorously washed in physiological solution (0.9% NaCl) for 10 min and surface-sterilized in 70% ethanol for 3 min, 1% sodium hypochlorite for 5 min, 70% ethanol for 30 s and rinsed four times for 2 min in sterile distilled water before a final washing step in sterile distilled water for 30 min. A 100 µL sample of the last rinsing water was plated on the same medium subsequently used for bacteria isolation, 1:10,869 medium [27], to confirm root surface sterility. Finally, roots were smashed with sterile mortar and pestle. One gram of the resulting root tissue homogenate was suspended in 9 mL of physiological solution, serially diluted and plated in triplicate on 1:10,869 medium prepared by using the 0.22 μm pore size filter-sterilized treated wastewater (TWW) obtained from the largest municipal wastewater treatment plant of Milan municipality (Milano-Nosedo, Northern Italy) and supplemented with cycloheximide 0.1 g/L to prevent fungal growth. Rhizosphere soil samples collected from the same specimens were pooled (1 g/specimen) and 1 gram of homogenized soil (fresh weight) was subjected to the same protocol described for the root tissue homogenate. After 48 hours of incubation at 30 °C, the number of colony-forming units (cfu) per gram of roots/soil was determined and, for each sample, bacteria colonies were randomly picked and spread three times on the same medium in order to obtain pure bacterial cultures [28]. A collection of 80 endophytic and 72 rhizospheric bacteria was established and cryopreserved in 25% glycerol stocks at −80 °C. The isolates were labeled with codes including sampling site (‘CWM’ for Constructed Wetland in Morocco), plants species (‘P’ for *P. australis*), medium used for the isolation (‘8’ for 1:10,869) and the fraction (‘R’/‘E’ for rhizosphere/endosphere) followed by progressive numbers.

### 2.3. Bacteria Genotyping and Identification

The genomic DNA of each isolate was extracted through boiling cell lysis [29]. The bacteria collection was de-replicated by fingerprinting analyses of the 16S–23S rRNA Intergenic Transcribed Spacer (ITS) region performing the ITS-PCR protocol described by Mapelli et al. [30]. The PCR products were separated on 1.5% agarose gel and ITS-fingerprinting profiles were visualized using Gel Doc system (Bio-Rad, Milan, Italy). Isolates which showed the same ITS band pattern were grouped in ITS clusters. At least one representative strain per each ITS cluster has been selected for subsequent physiological characterizations and for taxonomic identification through 16S rRNA gene amplification using the universal primers 27F and 1492R as described by Mapelli et al. [30]. Partial 16S rRNA sequences were obtained from Macrogen, Rep. of South Korea. Nucleotide sequences were edited in Chromas Lite 2.01 and compared with those deposited in the GenBank database, using the BLAST suite. The partial 16S rRNA gene sequences of the bacterial isolates were deposited in the EBI database under the accession numbers LS991341-LS991401.

### 2.4. In Vitro Screening of Plant Growth Promoting Activities and Rhizocompetence Potential

The screening of Plant Growth Promoting (PGP) traits in the bacterial isolates was focused on biostimulation and rhizocompetence-related activities. For biostimulation indolacetic acid (IAA) production was assessed following the protocol described by Bric et al. [31] and the ACC-deaminase (ACC-d) activity was determined by the method of Penrose and Glick [32] using Salt Mineral medium supplemented with ACC as unique N source. Protease activity was determined from clearing zones in skimmed milk agar according to Nielsen and Sørensen [33]. The rhizocompetence potential of the isolates was established by applying different in vitro analyses. First, the production of exopolysaccharides (EPS) was evaluated according to Santaella et al. [34] using modified Weaver mineral media enriched with sucrose. Swimming and swarming lifestyles were considered to evaluate bacterial mobile pattern using solid medium prepared with 0.3% and 0.5% of agar according to Mi et al. [35]. The biofilm production capacity of the isolates was evaluated by a colorimetric assay based on crystal violet staining, as described below [36]. Two-hundred µL of bacterial suspension at 10^6^ cell/ml were added in 96-wells microtiter and after 24 h of incubation the optical density (OD) was measured at 610 nm using a microtiter-plate reader (Tecan Infinite F200Pro). The supernatant was removed, the wells were washed twice with 200 µL of PBS and let dry for 15 min before the staining with 200 µL of crystal violet (diluted 1:10 with EtOH) for 15 min. The wells were then washed twice with distilled water and let dry for 15 min. Crystal violet was re-suspended with EtOH 97% and OD was measured at 610 nm. The OD measure was compared with the absorbance of *E. coli* ATCC25404 used as positive control and the quantity of formed biofilm was expressed as percentage. Each assay was performed in triplicate. The non inoculated medium was stained as well with crystal violet and used as negative control to determine background OD.

### 2.5. Plant Growth Promotion of Model Plants Under Liquid Substrate Culture and of Juncus acutus Plants in Soil

Bacterial isolates were grown in Tryptic Soy Broth (TSB) for 24 h at 30 °C, the bacterial cells were centrifuged at 4000 rpm for 15 min and the pellet was re-suspended with 10 mM MgSO_4_ to obtain a bacterial concentration of 10^8^ cell/mL. *Lycopersicon esculentum* seeds, chosen as model plant, were sterilized with 70% ethanol for 3 min and then 5% sodium hypochlorite for 10 min followed by five rinsing steps in sterile distilled water. Surface-sterilized seeds were soaked for 1 h in the bacterial suspension at a concentration of 10^8^ cell/ml; non-inoculated seeds were watered with sterile distilled water as negative control. Treated seeds were transferred to germination pouches (CYG Seed Germination Pouches, Mega International, Minneapolis) under sterile condition (seven seeds per pouch). Five pouches per each bacterial strain/negative control were established. The pouches were watered with 20 mL of sterile tap water and placed under greenhouse conditions. After 20 days, different morphometric parameters of plants were recorded: seed germination, root length, shoot length, secondary root development and vigor index (i.e., % of germination X seedling length) of the seedlings.

Plant growth promoting test was also performed with *J. acutus* plants in soil under greenhouse conditions. Four seedlings of the same weight were bacterized with a mixture of the six most promising strains (10^8^ cell/mL) and four non-inoculated plants were used as negative control. After 45 days of growth, root and shoot lengths, root and shoot fresh and dry biomasses were measured; chlorophyll leaf content was measured according to Sharma et al. [37] method. All original data related to the in vivo PGP tests reported in this work are available within the Dataverse ‘madforwater-wp2’ created by the University of Milan at the following link: https://doi.org/10.13130/RD_UNIMI/VXKHSF.

### 2.6. Bacterial Tolerance to Metals and Emerging Organic Pollutants (EOP)

The isolates were tested for their ability to grow in the presence of increasing concentration of CdCl_2_ (0.05, 0.5 and 1 mM), ZnCl_2_ and NiCl_2_ (0.5, 1 and 2 mM) and all the three metals mixed together at two different concentrations (CdCl_2_-ZnCl_2_-NiCl_2_: 0.1 µM-30 µM-1.7 µM; 0.5 µM-150 µM-8.5 µM). The bacterial ability to tolerate metals was investigated in a 96 wells-microtiter with 180 µL of TSB medium (with and without metals) and 20 µL of bacterial culture grown until the late exponential phase. Each strain was tested in triplicate. The absorbance at 610 nm was measured with a microtiter-plate reader (Tecan InfiniteF200Pro) at time zero and after 24 and 48 h of incubation at 30 °C. Bacterial tolerance to Bisphenol-A (BPA) was also evaluated. After bacterial cultivation in TSB medium, 1 mL of culture broth was centrifuged at 4000 rpm for 15 min. The cell pellet was washed three times with sterile 10 mM MgSO_4_ and 100 µL of bacterial culture were spread on 284 solid medium [25] with or without the supplement of 100 µL of 100 mg/L BPA spread on the plate as a sole carbon source. The plates were incubated at 30 °C for 7 days. Antibiotic susceptibility test of the strains was performed with a disk-diffusion assay using 6 different antibiotics (the molecules were selected to span several mechanisms of action): cephalothin (30 µg), chloramphenicol (30 µg), ciprofloxacin (5 µg), rifampicin (5 µg), tetracycline (30 µg) and vancomycin (30 µg) (LABORATORIOS CONDA S.A., Madrid, Spain). After 24 h of incubation at 30 °C, bacteria were classified as sensitive or resistant according to the interpretative standards provided by LABORATORIOS CONDA S.A. using *E. coli* ATCC 25,922 as reference strain.

### 2.7. In Vitro Study of Bacterial Dye Decolorization Potential

Reactive Black 5 (dye content ≥ 50%, Sigma-Aldrich, RB5) is the commonly used azo-dye chosen as model molecule to examine bacterial decolorization capability [3]. Rhizospheric strains were thus tested for RB5 decolorization capacity, while a selection of these rhizospheric strains was also tested for the capacity to decolorize different dyes used by a textile company located in Tunisia: Bezactive rouge S-Matrix, Tubantin blue and Blue S-2G. Bacterial inocula were added to TSB liquid medium containing 100 mg/L of dye. At time zero and after 72 h of incubation at 30 °C, 1 mL of the culture broth was centrifuged at 8000× *g* for 5 min and the absorbance of the supernatant was measured using a spectrophotometer (597 nm for RB5, 522 nm for Bezactive rouge S-Matrix, 589 nm for Tubantin blue and 615 nm for Blue S-2G). Each strain was tested in triplicate and a non-inoculated negative control was assessed. Decolorization efficiency was calculated, according to Wang et al. [4], as:

$$\mathrm{Decolorization}\;\mathrm{efficiency}\;(\%)=\frac{{\mathrm{OD}}_{0}-{\mathrm{OD}}_{1}}{{\mathrm{OD}}_{0}}\times 100$$

where OD_0_ referred to the initial absorbance and OD_1_ referred to the absorbance after 72 h of incubation. The absorbance value was then related to dye concentration according to the standard curve obtained with known dye concentrations ranging from 2.5 to 150 mg/L.

### 2.8. Bioaugmentation of Juncus acutus Microcosms for the Treatment of Mixed Contamination

Plants of *J. acutus* were collected from Souda bay (Chania, Crete, Greece) and washed with freshwater. The ability of *J. acutus* to grow in presence of single and mixed contamination was estimated under greenhouse conditions in glass vessels covered with aluminum foil and filled with 950 g of gravel to support the plant (around 25 g). During this first experiment, four conditions were setup in 350 ml-replicate vessels (n = 3), differing in the liquid phase content: (a) RB5 50 mg/L; (b) RB5 10 mg/L + metals mixture (CdCl_2_-ZnCl_2_-NiCl_2_: 0.5 µM-150 µM-8.5 µM); (c) RB5 5 mg/L + metals mixture (CdCl_2_-ZnCl_2_-NiCl_2_: 0.5 µM-150 µM-8.5 µM); (d) tap water as control. After three weeks of growth, the health status of the plants irrigated with the contaminated solutions was compared to that of the control plants, assessing the capacity of *J. acutus* to cope with the dye and metal presence.

**Figure 1:**
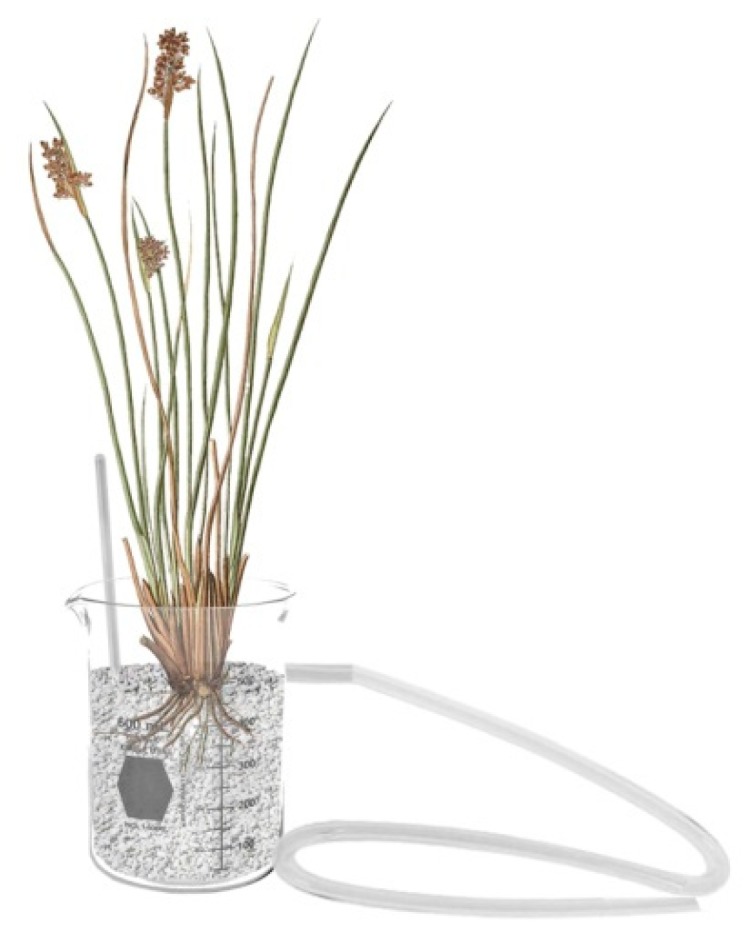
Schematic representation of the CW-microcosm with *J. acutus* plant and gravel as substrate. The pipette on the left allows to introduce the contaminated irrigation solution into the beaker and the tube on the right allows to collect the microcosm effluent for further analyses.

After this procedure, a new experiment was setup using six bacterial strains as single inoculum (10^8^ cell/mL) and as a consortium with *J. acutus* plants (around 19 g) growing in gravel as substrate (390 g) in modified beakers (Figure 1). Three replicated microcosms per each bacterial treatment were prepared. The control microcosms were setup with *J. acutus* plants without bacterial inoculation. Each microcosm was filled with 150 mL of a solution containing RB5 10 mg/L and metals (CdCl_2_-ZnCl_2_-NiCl_2_: 0.5 µM-150 µM-8.5 µM). In order to simulate the operational conditions of a constructed wetland system, every day the microcosms were irrigated with 25 mL of the solution described above, freshly prepared, and the effluents were collected measuring the concentration of RB5 (as explained above). After 27 days of operation the plants were harvested and the fresh and dry weight of *J. acutus* roots and shoots were measured to determine the in vivo PGP ability of the strains.

## 3. Results

### 3.1. Cultivable Bacteria Associated to Phragmites australis in Constructed Wetlands Treating Municipal Wastewater

A collection of 80 endophytic and 72 rhizospheric bacteria was obtained from *P. australis* surface-sterilized roots and rhizospheric soil, respectively. The abundance of cultivable bacteria was similar in the rhizosphere and in the endosphere fractions: 2.5 × 10^8^ ± 4.6 × 10^7^ and 1.2 × 10^8^ ± 4.4 × 10^7^ cfu per gram of fresh soil/root respectively.

**Figure 2:**
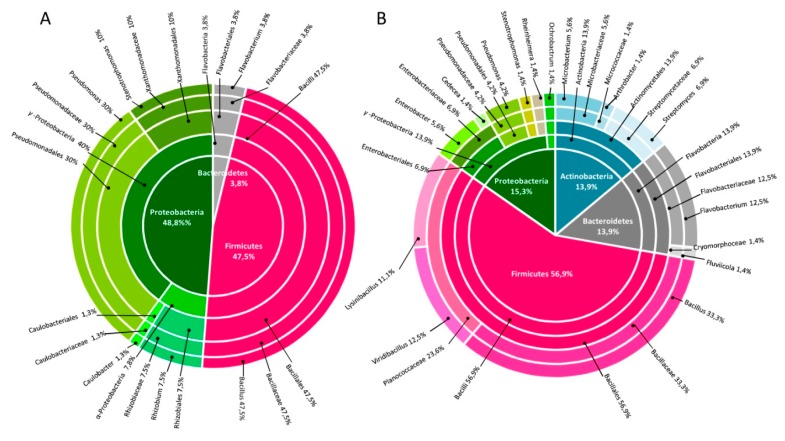
Taxonomic distribution of 16S rRNA sequences of culturable endophytic (**A**) and rhizospheric (**B**) bacteria isolated from *P. australis* plants. The inner pie graph shows the strain taxonomic identification according to phylum and each outer ring represents their identity at the class, order, family and genus level.

The ITS fingerprinting analysis was applied to de-replicate the bacterial collection and reduce its phylogenetic redundancy. Forty-five ITS profiles were recognized within the rhizospheric isolates and 16 ITS profiles within the endospheric ones. The strains, identified by partial 16S rRNA sequencing, belonged to 4 phyla, namely *Actinobacteria*, *Bacteroidetes*, *Firmicutes* and *Proteobacteria* (Supplementary Table S1). The most abundant genera in the overall collection were *Bacillus* and *Pseudomonas*, representing 41% and 18% of the strains, respectively. Among the two fractions of the *P. australis* root system, endosphere and rhizosphere, we nevertheless observed a different distribution of the bacterial phylogenetic groups (Figure 2A). The endophytic strains showed reduced phylogenetic diversity, belonging only to six genera. 78% of the endophytic strains were assigned to the genera *Bacillus* and *Pseudomonas*: 89% of the *Bacillus* isolates belonged to *B. pumilus* and the 71% of the *Pseudomonas* isolates was identified as *P. thivervalensis*. Thus, a limited and peculiar community colonized the endosphere fraction of *P. australis*. In contrast, the rhizosphere fraction showed a higher bacterial diversity. 57% of the rhizosphere strains were grouped within the *Firmicutes* phylum while the other isolate strains of the rhizosphere were equally distributed among *Actinobacteria* (14%), *Bacteroidetes* (14%) and *Proteobacteria* (15%) phyla (Figure 2B). Apart from the species *B. thuringiensis*, that included the 24% of the rhizobacteria isolates, the other species present in the rhizosphere were represented by few or single isolates, which explains the high bacterial variability into this fraction.

Only 8 endophytic isolates were considered as potentially pathogens for humans and plants according to their taxonomy and to a reference document provided by the German Committee on Biological Agents that classifies Prokaryotes into Risk Groups [38]: these potentially pathogens isolates belonged to the species *Pseudomonas aeruginosa* and *Agrobacterium tumefaciens* (Supplementary Table S1) and were excluded from the collection given their low suitability for future applications in the field linked to safety issues.

### 3.2. PGP Potential of P. australis Associated Bacteria

**Figure 3:**
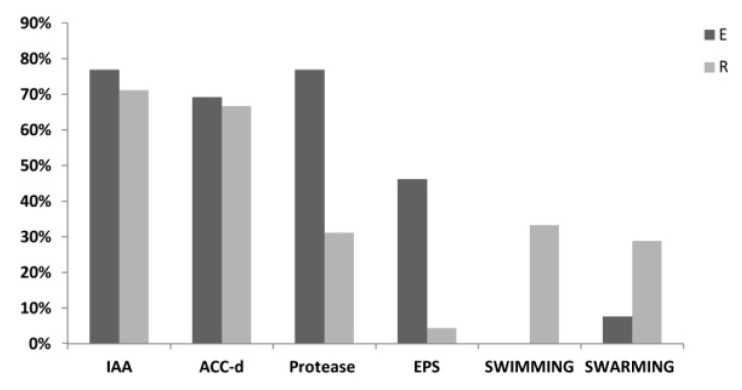
Characterization of the bacteria collection for plant growth promotion (PGP) traits. Percentage of strains isolated from endosphere (E, black) and rhizosphere (R, gray) fractions that resulted positive to PGP assays. Among PGP activities: IAA = indole-3-acetic acid production, ACC-d = ACC deaminase activity, Protease = protease production, EPS = exopolysaccharides production, SWIMMING = swimming lifestyle, SWARMING = swarming lifestyle.

The potential of the bacterial strains associated to *P. australis* to sustain plant growth was assessed by screening in vitro the PGP traits potentially involved in root development, a key aspect for CW plants, which are threatened by different abiotic stresses [39]. In particular, we assessed whether the isolates were able to produce indolacetic acid (IAA), a phytohormone that promotes the root apparatus growth [40], and whether they displayed ACC-deaminase activity that potentially reduces the deleterious effect of ethylene, the plant stress-related hormone, by degrading its precursor ACC [41]. 72% of the strains were able to produce IAA and 67% showed ACC-deaminase activity, while 53% showed to have both the activities. Despite the difference in phylogenetic composition, these two PGP-related traits resulted similarly represented among endosphere and rhizosphere isolates (Figure 3). Moreover, the isolate collection was tested for additional traits that could sustain the capacity of the strains to degrade WW organic material and to move into the environment and adhere to the root surface. In total, 41% of the strains were positive to protease activity test and 14% of the isolates produced EPS, mainly represented by endophytes. The swimming and swarming lifestyle (Figure 3) was shown by 26% and 24% of the isolates, respectively, most of which belonging to the *Firmicutes* phylum, and it was more widespread among the rhizosphere strains. *Bacillus*, *Lysibacillus* and *Pseudomonas* showed to predominantly hold these PGP traits among the different genera (Supplementary Table S2).

The PGP capability of *P. australis*-associated bacteria was also tested in vivo under liquid substrate culture using *L. esculentum* as model plant bacterized on seeds (Supplementary Table S4). Thirty-nine of the 58 tested isolates (3 endophytic and 36 rhizospheric strains) showed significant growth promotion in tomato inoculated plants in comparison to non-inoculated ones. Among the different measured parameters, the most positively affected by the bacteria inoculation were the shoot length (Supplementary Figure S1) and the vigor index of the seedling: 31 bacteria significantly increased shoot length and 23 bacteria improved the seedling vigor index. Moreover, 18 strains improved root length, 7 different rhizobacteria enhanced the root dry weight and other 5 strains enhanced the number of secondary roots. Finally, 5 strains increased the percentage of germination. Most of the bacteria with PGP capability on tomato plants improved at least two physiological parameters and the strains *Viridibacillus arenosi* CWMP-8R10 and *Bacillus megaterium* CWMP-8R7 enhanced five out of the six assessed parameters.

### 3.3. Metal and Organic Pollutant Tolerance and Degradation Potential of the Isolated Bacteria

**Figure 4:**
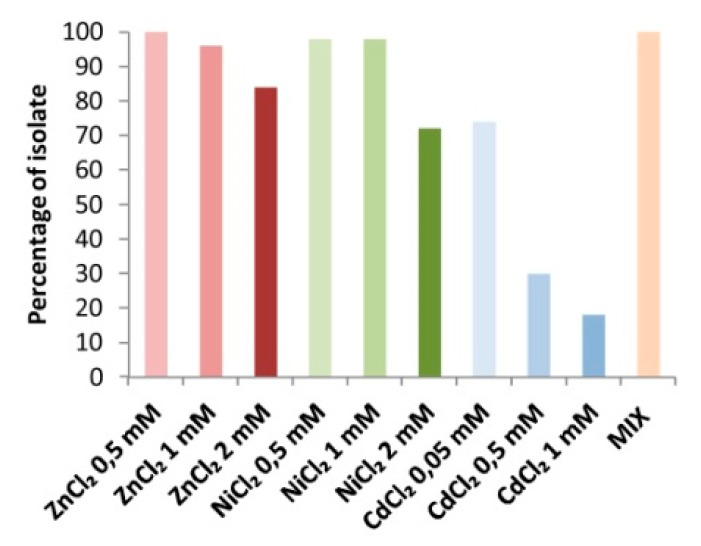
Characterization of the bacteria collection for metal tolerance. Percentage of strains tolerant to ZnCl_2_, NiCl_2_ and CdCl_2_ at three different concentrations and to the MIX of metals (30 µM ZnCl_2_, 1.7 µM NiCl_2_ and 0.1 µM CdCl_2_).

The bacterial collection proved to include a high number of metal-resistance strains. Among the tested isolates, 15 different metal-resistance phenotypes were detected (Supplementary Table S3). All the strains were able to grow on rich medium containing the three metals in micromolar concentrations supplemented simultaneously (ZnCl_2_, NiCl_2_ and CdCl_2_, 30 µM, 1.7 µM and 0.1 µM respectively) (Figure 4) and 12% of the isolates were tolerant to the three metals (Cd, Ni and Zn) at all the tested concentrations, up to 1–2 mM. Τhe large majority of the isolates resulted tolerant to Zn and Ni, while on the contrary, the sensitivity to Cd was very high and only 30% of the strains was able to grow in a medium containing this metal at the lowest concentration (0.5 mM CdCl_2_) (Figure 4). The tolerance to CdCl_2_ was further assessed at a concentration lower than 0.5 mM, allowing the identification of strains tolerant to 0.05 mM CdCl_2_ (74% of the collection). 

**Figure 5:**
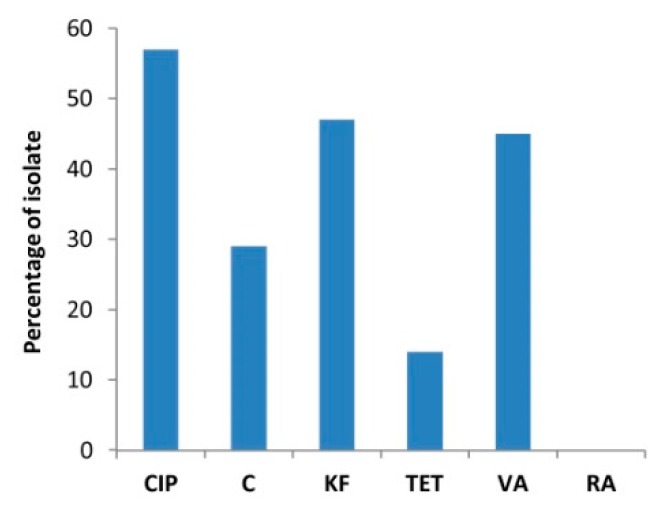
Characterization of the bacteria collection for antibiotic resistance. Percentage of strains resistant to CIP = ciprofloxacin (5 µg); C = chloramphenicol (30 µg); KF = cephalotin (30 µg); TET = tetracycline (30 µg); VA = vancomycin (30 µg); RA = rifampicin (5 µg).

The ability of the strains to tolerate organic pollutants was assessed on model micropollutants typically found in municipal and industrial wastewaters like BPA and antibiotics. All the rhizospheric bacteria and 77% of the endophytic bacteria showed tolerance to 100 mg/L BPA supplemented as unique carbon source to the growth medium (Supplementary Table S4, Table 1). However, these bacteria grew also on control plates with mineral medium and no addition of BPA, so probably they tolerated BPA presence, but they do not have the ability to use it as carbon source. Antibiotic resistance was also widespread within the bacterial collection, since only 24% of the isolates were inhibited by all the tested antibiotics. None of the strains were resistant to rifampicin, but the resistance to the other antibiotics was detected in the majority of the strains. In particular, the resistance to ciprofloxacin, cephalotin and vancomycin was observed in 57%, 47% and 45% of the strains among the bacterial collection, respectively (Figure 5). Most of the isolates showed multi antibiotic-resistance phenotype and among the tested isolates, 17 different antibiotic-resistance phenotypes were detected: as shown is Table 2, 2% of the bacterial isolates was resistant to five of the tested six antibiotics and 19% of the isolates showed four or three resistance simultaneously.

The rhizobacteria collection (n = 36) was tested for the ability to decolorize the model azo-dye molecule Reactive Black 5 (Supplementary Table S4; Table S2). We tested specifically rhizobacteria for this activity, considering that the decolorization of azo-dye contaminated wastewaters in CW systems should be more significant in plant rhizosphere rather than in the endosphere. The results of the decolorization assay clustered the isolates in three groups according to their dye decolorization efficiency that was higher than 50% (17% of the tested strains), comprised between 20–50% (33% of the tested strains) and lower than 20% (50% of the tested strains).

### 3.4. Selection and Characterization of the Most Promising Strains for Bacterial Enhanced Phytodepuration

Within the bacteria collection, six strains were identified as the most promising for future application in phytodepuration systems, basing on the in vitro and in vivo characterization in terms of PGP activity, BPA tolerance and RB5 decolorization activity (Supplementary Table S4; Table S2). For the bacterial selection we also attempted to choose bacteria that presented resistance to few antibiotic molecules, as resulted by disk-diffusion assays. Given that the majority of the isolates of the collection was resistant at least to one of the tested antibiotics under our experimental conditions, the selection was forced to include, among the bacteria with best PGP properties, BPA and metal tolerance and RB5 decolorization ability, those resistant to few antibiotics. The physiological features of the six selected strains (*Pseudomonas fluorescens* CWMP-8R25, *Microbacterium oxydans* CWMP-8R34, *Microbacterium maritypicum* CWMP-8R67, *Flavobacterium johnsoniae* CWMP-8R71, *Lysinibacillus fusiformis* CWMP-8R75 and *Enterobacter ludwigii* CWMP-8R78) are summarized in Table 1. These bacteria were further analyzed for other abilities important for the possible exploitation in bioaugmentation approaches, such as the capability to form biofilm and adhere to the plant root apparatus and their versatility in terms of tolerance/degradation of pollutants. The six strains were tested for the biofilm formation ability and 3 of them showed a biofilm formation higher than 50% compared to the *E. coli* ATCC25404 reference strain, one strain had a percentage of 44% and the remaining 2 strains showed a percentage lower than 20% (Table 1). All the strains were tolerant to a mixed solution of metals (CdCl_2_-ZnCl_2_-NiCl_2_: 0.5 µM-150 µM-8.5 µM) five times more concentrated than that used to screen the whole bacteria collection. Finally, the decolorization of additional synthetic dyes (Bezactive rouge S-Matrix, Tubantin blue and Blue S-2G) was assessed and although most of the bacteria showed a low dye decolorization efficiency (<20%), the two strains *F. johnsoniae* CWMP-8R71 and *E. ludwigii* CWMP-8R78 revealed a high efficiency of decolorization for all the tested dyes (Table 1). 

### 3.5. Evaluation of the Bacterial Contribution to Azo-Dye Removal by Juncus acutus in Microcosm-Scale CWs

**Figure 6:**
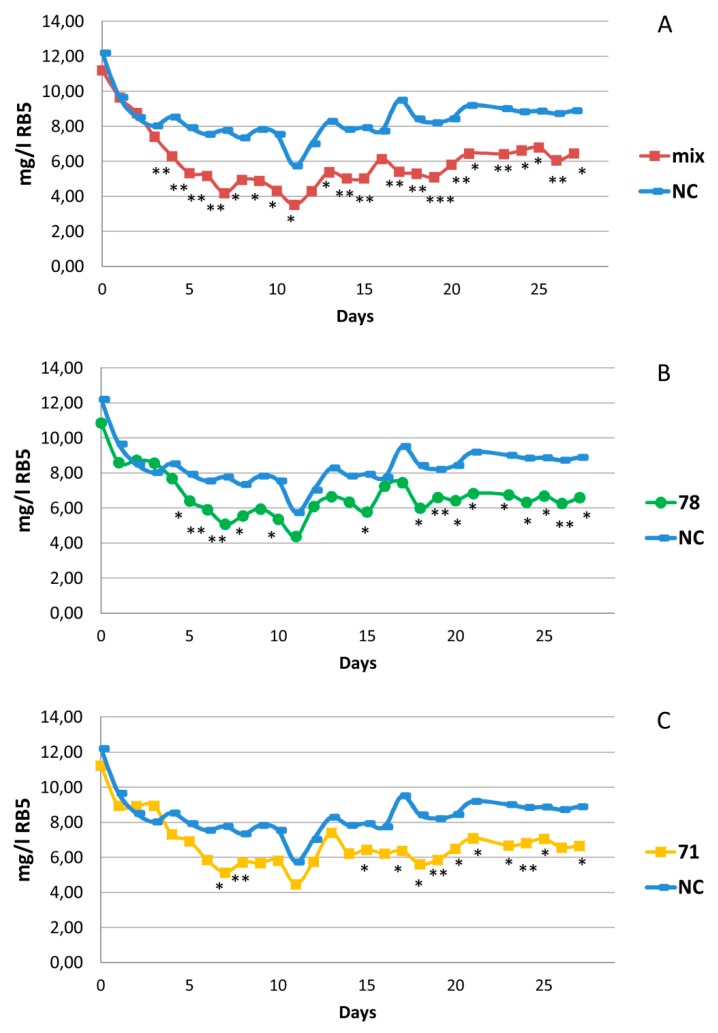
Concentration of RB5 daily measured from microcosm effluents over the experimental period (27 days). Values are reported as a means of the replicated CW microcosms (n = 3). RB5 concentration was compared between (**A**) NC = negative control, non-bacterized *J. acutus* plants, and plant inoculated using a consortium of the six isolates (mix); (**B**) NC and plants inoculated with 78 = *E. ludwigii* CWMP-8R78; (**C**) NC and plants inoculated with 71 = *F. jonhsoniae* CWMP-8R71. The stars indicate the days were statistically significant differences were observed (* *p* < 0.05, ** 0.001 < *p* < 0.01, *** *p* < 0.001).

A preliminary greenhouse experiment was setup to assess if *J. acutus* plants grown in a solution containing the azo-dye RB5, alone or in combination with metals, were able to tolerate the contaminants. The plants were able to grow under the setup conditions without showing any visible inhibition in comparison with control plants irrigated with tap water. A similar biomass amount of *J. acutus* plants was subsequently used to set up microcosms bacterized and artificially irrigated with a solution containing the RB5 azo-dye with the presence of metals, aiming to provide a stress factor to the plants (Figure 1). Triplicate microcosms were set up for each assay, represented by the single inoculum of the six selected strains, and a consortium including all the six bacteria together in equal concentrations. The amount of RB5, added daily to the microcosms with 25 mL of inflow containing 10 mg/L of dye, was monitored in the microcosm effluents for an overall period of 27 days and at the end of the experiment plant biomass was measured. None of the strains showed the promotion of root and shoot growth of *J. acutus* plants (data not shown) while positive results were obtained for two strains and the six-bacteria consortium in terms of RB5 decolorization (Figure 6). The RB5 concentration in the effluent of all the microcosms showed high fluctuations, which decreased over time. After 19 days the systems reached a stability condition, since the percentage of variation of RB5 concentration in the effluents in consecutive days was reduced to ± 10%. The results of the CW bioaugmentation experiment revealed that the RB5 concentration decreased also in the effluents of the non-inoculated *J. acutus* microcosms (Figure 6) indicating the depuration potential of the plant itself. However, the use of *F. johnsoniae* CWMP-8R71, *E. ludwigii* CWMP-8R78 and the consortium of the six bacteria (MIX) proved to be effective as inoculum for the bioaugmentation of *J. acutus*, given the higher decolorization efficiency observed in the treated CW microcosms compared to the control microcosms which did not received the bacteria inoculum (Figure 6A–C). We considered that the bacterisation significantly improved RB5 removal when the difference in its concentration between bacterized and not bacterized microcosms was statistically significant in more than 85% of the days in the stability time frame (i.e., after day 19). In particular, we observed a rapid response of the consortium, which, two days after the bacterization of *J. acutus* plants, was already able to significantly improve the decolorization of RB5 in comparison to the non- inoculated plants. Moreover, the positive result showed by the consortium, was more stable throughout the whole experimental time, significant in 100% of the days after system stabilization, while the six different single inocula showed higher fluctuations. Even if in microcosm conditions the consortium did not significantly affect *J. acutus* growth, in the soil experiment it showed a significant promotion of root biomasses and chlorophyll leaf content (Supplementary Table S5).

## 4. Discussion

The selection of bacterial strains exploitable to improve phytodepuration should be the result of a broad screening that, starting from a large bacteria collection isolated from the root apparatus of the plant of interest, considers: i) the phylogenetic classification of the strain, in order to discharge possible human, animal and plant pathogens, ii) the characterization of the PGP potential of the isolates in vitro and in vivo on model plants, iii) the evaluation of the bacterial tolerance to different micropollutants which are typically found in WWs and could, in principle, contribute to create a stressful environment for the plant and the associated bacteria, and iv) the evaluation of the bacterial ability to degrade the pollutant of interest. In this frame, we applied a cultivation approach to isolate bacteria from root endosphere and rhizosphere of *P. australis*, the plant species most exploited in phytodepuration processes [20]. A high diversity in terms of bacterial taxonomy and abundance was found between the rhizosphere and endosphere fractions, as already reported in other studies and in different plant species [26,42]. Plants recruit in the rhizosphere a specific microbiome from the soil microbial community through the release of rhizodepositions, and only a selected number of microbes are able to overcome the root barrier and establish in the endosphere [43]. The results of this work, even if obtained on a single composite sample and only on the cultivable fraction of the bacterial community, demonstrated clearly this selection effect. Rhizosphere and endosphere collections differed substantially in terms of diversity with 14 genera isolated in the rhizosphere and only 6 in the endosphere. The two plant niches shared 4 bacterial genera, which were present nevertheless in different percentages. The bacteria collection obtained from *P. australis* endosphere was mainly represented by the genera *Bacillus* (48%) and *Pseudomonas* (30%), in agreement with previous reports. Shehzadi et al. [44] showed the abundance of the genus *Bacillus* in the endosphere of different wetland plant species, whereas several studies confirmed the *Pseudomonas* prevalence in the endosphere of *P. australis* [45,46,47]. The *Bacillus* and *Pseudomonas* genera were commonly found associated to the root apparatus of plants growing under adverse environmental condition [24,48], since they are able to use a wide range of substrates as energy and carbon sources and are often tolerant to toxic compounds [46,49]. The strain collection obtained from *P. australis* rhizosphere in this study was dominated by *Firmicutes* (57% of the isolates), with a lower percentage of *Proteobacteria* and *Bacteroidetes,* that found a more suitable habitat in the plant endosphere, and *Actinobacteria*, that conversely were not isolated from the endosphere fraction. Previous studies, in contrast with our results, by applying cultivation-independent Next Generation Sequencing approaches retrieved in the rhizosphere of *P. australis* the dominance of the phylum *Proteobacteria* [50,51,52]. The dominance of *Firmicutes* in our collection could be a cultivation-related bias, although we have to consider that the plant species is not the only factor that modulates the bacterial community structure in the root apparatus, acting together with different abiotic factors [53] and making reasonable that the rhizosphere community of *P. australis* collected from CW systems treating different wastewaters hosts specific peculiar gram positive taxa. A further study of the bacterial community structure evaluated by metataxonomic approaches would lead to obtain a clearer picture about the existence of a core phylogenetic composition in the *P. australis* microbiome. Bacterial isolates are nevertheless necessary in order to exploit them for CW bioagumentation. 

The root apparatus of *P. australis* growing in a CW treating municipal wastewaters may harbor different pathogenic microorganisms, unsuitable for their future exploitation as inoculants. Differently from Calheiros et al. [45], which isolated a high number of putative human pathogens from the endosphere of *Canna flaccida* plants grown in a CW system, basing on strain phylogenetic identification we found in our collection only few putative animal/human pathogenic strains, phylogenetically related to the *P. aeruginosa* species and present only in the plant endosphere. It is known that one of the services provided by CW depuration systems is the high removal rates of enteric and fecal indicators [54], but the efficiency may depend on different operational parameters, including also plant species and plant permeability, justifying the differences observed in the two studies. The strains identified as the human potential pathogen *P. aeruginosa*, together with the plant potential pathogen *A. tumefaciens*, were no longer taken into account for the following analysis aimed at selecting bacterial strains exploitable in the improvement of CW performances.

In order to identify putative inoculants to be used in CW systems, we investigated our bacterial collection for the ability to promote plant growth. The PGP potential of the strains was assessed in vitro focusing on biostimulation related traits (i.e., ACC-deaminase activity and IAA production), relevant to sustain plant growth under the abiotic stresses that plants have to deal with in CWs, like saline waters, organic load fluctuation, presence of micropollutants [55,56]. Bacterial mediated modulation of plant hormonal balance has the potential to reduce the plant perception of stress conditions and to promote the development of the root apparatus [40,41]. Root biomass improvement is of paramount importance for the macrophytes used in CW systems since it has the potential to result in an improved filtration and reduced flow velocity of wastewaters, besides offering a higher surface and more extended habitat for the colonizing microbial community [57]. As shown in other studies performed on *P. australis*, a high percentage of bacteria isolated from both endosphere and rhizosphere was able to produce IAA and exhibited ACC deaminase activity [47,52], showing a significant potential of *P. australis* microbiome in sustaining plant adaptation and growth under the stressed conditions of a CW system.

Forty-one percent of the isolates exhibited, moreover, protease activity, an interesting trait for bacteria involved in the process of wastewater phytodepuration by catalyzing proteolysis that is the first degradation step of organic nitrogen compounds. Navarro-Torre and coworkers [58] discussed about the importance of the enzymatic activity potential and showed that protease activity was one of the most common enzymatic activities within the bacterial collection isolated from a halophyte plant naturally growing in marshes and potentially useful for phytoremediation purposes because of its heavy metal-tolerance.

The PGP potential of the isolates was additionally tested in vivo under hydroponic conditions using tomato as model plant. Madhaiyan et al. [59] used the same system to prove the PGP potential of two bacteria isolated from rice, resulting able to promote the root elongation of *L. esculentum*. Our results confirmed the high PGP potential of strains recruited by *P. australis*, in particular the rhizospheric ones, which resulted more phylogenetically diverse and more active in the promotion of growth traits since 80% were able to promote at least one of the measured parameters. The results obtained in this work on *P. australis* microbiome confirm previous studies that demonstrated that plants adapted to stressed and contaminated environments represent an excellent reservoir of bacterial strains with multiple PGP capabilities able to enhance the host plant stress-tolerance and growth [44,48,60,61].

All the rhizospheric strains have been tested for their ability to decolorize RB5 azo-dye: the best RB5-performing ones were then screened for the decolorization of other additional colorants commonly used by the textile industry (Bezactive rouge S-Matrix, Tubantin blue and Blue S-2G). Azo-dyes are the most common synthetic organic dyes used in textile industry and, being released through the textile industry effluents, they represent wide spread xenobiotic pollutants. Even if we isolated the bacteria from plants collected in a municipal wastewater CW not specifically polluted by textile effluents, we found that 17% of the tested isolates showed significant RB5-decolorization with efficiencies higher than 50%, while 33% had decolorization efficiency between 20 and 50%.

The ability of the strains to tolerate different model micropollutants completed the strain selection for the subsequent microcosm-based tests, given the high variety of chemical compounds that can be found in sewages and could constitute stress factors for plants and bacteria. We focused in particular on BPA, employed as a primary raw material for the production of poly-carbonate plastics but also for flame retardants used in textile industry [62] and used as intermediate chemical in the manufacture of antioxidants and dyes [63]; moreover we looked for metal tolerance since they are always found into the textile industry effluent as micropollutants [1,64,65]. Ninety-five percent of the isolates were tolerant to 100 mg/L BPA present in the growth medium. A similar result was reported by Syranidou et al. [25] studying a collection of endophytic bacteria isolated from *J. acutus* roots and leaves. A high percentage of the strains associated to *P. australis* possessed also high tolerance to metals like Zn, Ni and Cd, both supplemented singularly or in mixture in the growth medium. Metal tolerance is a useful trait in CW macrophyte plant associated bacteria since they could enhance the plant metal uptake and their translocation to the aboveground tissues [66].

Syranidou et al. [67], working with endophytic bacteria isolated from *J. acutus*, observed that the leaf isolates were particularly dominated by metal tolerant strains, although this plant shows a tendency to accumulate metals in the belowground tissues. Our results demonstrated that in *P. australis* metal tolerant strains are present both in root rhizosphere and endosphere. According to Syranidou et al. [67] among the tested metals the tolerance to cadmium was less common than to zinc and nickel, in particular among the endophytic bacteria, where the majority of the strains were tolerant to cadmium only at low concentration. This could be related to a differential phytoextraction ability of this plant species that should be further evaluated.

To identify the most promising isolates for the inoculation of macrophytes in a CW plant, we screened the collection also for the swimming/swarming ability and EPS production, which are important traits improving the competitiveness of inoculants and thus indicating a root colonizing competence. Specifically, only 26% and 24% of the tested isolates showed swimming and swarming lifestyle, respectively, likely because these motility lifestyles are typically dependent on stringent growth conditions [68] which cannot be generally found in wastewaters. Moreover, swarming motility has been currently verified in *Gamma/Alpha-Proteobacteria* and *Firmicutes* phyla [69]: the majority of our strains with swarming motility were indeed identified as *Firmicutes*. The few bacteria of our collection that produced EPS were all isolated from the plant endosphere even if other works showed that also rhizobacteria have this ability, e.g., bacteria isolated from *Salicornia strobilacea* rhizosphere [48] and from *P. australis* rhizosphere growing in Cu-polluted conditions [70].

Antibiotics are widespread micropollutants in the human-impacted environments, especially in municipal WWs [71]. Most of the *P. australis* isolates showed to tolerate the presence of one or more antibiotics when supplemented in the growth medium, demonstrating that they have the potential to be active even in CWs receiving antibiotic contaminated influents. Our selection of the most promising bacteria to be exploited as CW inoculants took nevertheless into account the strain antibiotic resistance profile, selecting among the PGP, azo-dye degrading and micropollutant tolerant isolates, those strains with the lowest levels of antibiotic resistance. This selection was made in compliance with the ‘One-Health’ approach aiming to minimize the environmental spread of antibiotic resistance genes. Antibiotic resistance was a highly common trait in our bacterial collection and, in agreement with the results of Mahfouz et al. [72], our isolates showed greater resistance to antibiotics that have been available for longer in the environment (e.g., ciprofloxacin, cephalotin and vancomycin).

Considering all the mentioned features and the ability of the isolates to decolorize RB5 in vitro, six strains (Table 1) were identified as the most promising CW inoculants aimed to the depuration of textile wastewaters. The isolates were used to inoculate *J. acutus*, a halophyte plant species exploited for wastewater treatment in constructed wetlands and able to tolerate a wide range of contaminants [73], which we demonstrated to cope with the presence of RB5 and metals. The phytodepuration of textile wastewaters polluted with azo-dyes was tested with other macrophyte genera like *Phragmites* and *Typha* [74,75,76,77] but never with *Juncus*. Among azo-dyes, RB5 is the most used and there are several studies focused on its removal from aqueous solutions through advanced methods such as electrochemical and anodic oxidation [77,78]. On the contrary, studies focused on the removal of RB5 with cheaper technologies like bacterial enhanced-phytodepuration have been mainly performed in vitro [2,4,79]. The bacterial performance to improve RB5 decolorization was tested for the first time in dynamic systems [56,67] by using as microcosm influent a solution containing metals and RB5 aiming to simulate the continuous flow conditions of a CW treating real textile wastewaters. Even if, during the experiment, the microcosms showed high fluctuations in terms of RB5 concentration in the effluent, putatively due to environmental variations (e.g., temperature, irradiation, humidity) that can daily alter water evaporation in the microcosms and plant evapotranspiration, the strains *Flavobacterium johnsoniae* CWMP-8R71, *Enterobacter ludwigii* CWMP-8R78 and the consortium of all the six inoculants significantly enhanced the phytodepuration potential of *J. acutus* plants when the system reached the removal stability. Both *F. johnsoniae* and *E. ludwigii* are recognized as efficient hydrocarbon degraders, plant growth promoters and good root colonizers [80,81,82], but no information about their azo-dye decolorization potential was provided by the literature. The six strains, when applied as pure culture to the plants did not improve the growth of *J. acutus* in CW microcosms during a 27 days treatment. However, the six-strain consortium showed appreciable PGP activity by significantly increasing root biomasses and the chlorophyll content of the leaves when applied for a longer period to potted soil *J. acutus* plants. The influence of the bacterial inoculation on the detoxification of textile effluent in a CW reactor planted with *Typha domingensis* was previously evaluated by Shehzadi et al. [83], proving the decolorization enhancement implemented by *Microbacterium arborescens* and *Bacillus pumilus* inocula. The effect of the inoculation of endophytic bacteria on the phytoremediation potential of *J. acutus* was also proved by Syranidou and coworkers [67] that showed the beneficial effect of bioaugmentation with *Sphingomonas* sp., *Bacillus* sp. and *Ochrobactrum* sp. in the removal of EOCs and metals from the liquid phase of the system.

The results of our study highlighted that the synergistic relationship between specific bacteria inoculants and plants enhanced *J. acutus* growth and CW services and could be exploited to design specific WWTPs oriented at the degradation of textile dyes, recalcitrant organic pollutants that are not degraded by other conventional aerobic water treatment plants [84].

## 5. Conclusions

This study reveals that the root apparatus of *P. australis* plants growing in CWs is naturally associated to a beneficial microbiome able to promote plant growth and to tolerate the presence of different classes of micropollutants commonly detected in municipal and industrial wastewaters. The obtained results allowed to identify in particular six most promising strains which, tested in CW microcosm-scale experiment, enhanced the azo-dye phytodepuration capacity of *J. acutus* plants. Even if the application of these strains did not result in a complete dye removal, they could anyway be further exploited for improving the treatment of textile industry effluents, with low cost sustainable systems of particular interest for the MENA region.

## Figures and Tables

**Table:** (**A**)

Strain	Closest Described Relative	IAA	ACC-d	Prot.	Hydroponic Experiment						Biofilm	Swimming	Swarming	EPS
					% Germin.	Root-l	Shoot-l	2ary Roots	SVI	Root-dw				
CWMP-8R25	*Pseudomonas fluorescens*	+	+			*	**	**	*		44%			
CWMP-8R34	*Microbacterium oxydans*	+	+				**	*			84%			
CWMP-8R67	*Microbacterium maritypicum*	+	+	+			***		*		52%			
CWMP-8R71	*Flavobacterium johnsoniae*					***	***		***		6%			
CWMP-8R75	*Lysinibacillus fusiformis*	+					***				66%	+	+	
CWMP-8R78	*Enterobacter ludwigii*	+	+			***	***		***		21%	+		

**Table:** (**B**)

Strain	Closest Described Relative	BPA	Antibiotic Resistance						Metal Tolerance (mM)											Decolorization			
			CIP	C	KF	TET	VA	RA	CdCl_2_			NiCl_2_			ZnCl_2_			MIX1	MIX2	RB5	BR	TB	BS2-G
									0.05	0.5	1	0.5	1	2	0.5	1	2						
CWMP-8R25	*Pseudomonas fluorescens*	+	R		R	R			+	+	+	+	+	+	+	+	+	+	+	68%	10%	0%	22%
CWMP-8R34	*Microbacterium oxydans*	+	R	R	R	R			+	+	+	+	+	+	+	+	+	+	+	71%	20%	7%	25%
CWMP-8R67	*Microbacterium maritypicum*	+	R			R			+	+	+	+	+	+	+	+	+	+	+	64%	21%	4%	13%
CWMP-8R71	*Flavobacterium johnsoniae*	+	R		R	R	R		+			+	+	+	+	+	+	+	+	74%	85%	58%	15%
CWMP-8R75	*Lysinibacillus fusiformis*	+	R						+			+	+	+	+	+	+	+	+	74%	39%	29%	15%
CWMP-8R78	*Enterobacter ludwigii*	+	R				R		+	+	+	+	+		+	+	+	+	+	27%	22%	35%	45%

**Table 2:** Antibiotic-resistance phenotypes. The code of the isolates is simplified and includes only the fraction of origin (R = rhizosphere; E = endosphere) and the progressive number. The percentage in the last column is referred to the strains belonging to each phenotype. CIP = ciprofloxacin; C = chloramphenicol; KF = cephalotin; TET = tetracycline; VA = vancomycin; RA = rifampicin. ‘R’: refers to the strain resistance to the antibiotic at the indicated concentration.

		Antibiotic Resistance (R)							
Phenotype	Code of the Isolates	CIP	C	KF	TET	VA	RA	N of R	% of Isolates
1	E13	R	R	R	R	R		5	2
2	R34, R52	R	R	R	R			4	19
3	E2, E8, E14, E28, E33, R32	R	R	R		R			
4	R12	R	R		R	R			
5	R71	R		R	R	R			
6	R20		R	R	R	R			
7	R25	R		R	R			3	19
8	E16, E42, E73, R65, R76, R79	R		R		R			
9	E6, E21, R4, R69		R	R		R			
10	R3	R		R				2	14
11	R67	R			R				
12	R17, R78	R				R			
13	R6, R28		R	R					
14	R22, R26			R		R			
15	E15, R2, R16, R23, R33, R39, R49, R50, R64, R75, R80	R						1	22
16	R8, R19					R			
17	E27, R1, R7, R9, R10, R15, R31, R38, R40, R47, R57, R68, R72, R77							0	24

## Supplementary Materials

Supplementary materials can be found at https://www.mdpi.com/2076-2607/7/10/384/s1.
