# Supplementary material for: Root Bacteria Recruited by Phragmites australis in Constructed Wetlands Have the Potential to Enhance Azo-Dye Phytodepuration
Source: Microorganisms. 2019 Sep 24;7(10):384. doi: 10.3390/microorganisms7100384 (PMC6843347; doi:10.3390/microorganisms7100384)

Supporting information of

Root bacteria recruited by *Phragmites australis* in Constructed Wetlands have the potential to enhance azo-dye phytodepuration

Supplementary Table S1. Distribution of cultivable bacteria isolated from *P. australis* roots and rhizosphere according to the phylogenetic classification at the phylum, genus and species level.

Phylum	Genus	E (80)	R (72)	Species	E (80)	R (72)
Actinobacteria	*Arthrobacter*		1	*A. ureafaciens*		1
	Microbacterium		4	*M. kitamiense*		2
				M. oxydans		1
				M. maritypicum		1
	Streptomyces		5	*S. pluricolorescens*		2
				S. althioticus		1
				S. acidiscabies		1
				S. caeruleatus		1
Bacteroidetes	*Flavobacterium*	3	9	*F. gyeonganense*	2	3
				F. johnsoniae	1	5
				F. oncorhynchi		1
	Fluviicola		1	*F. taffensis*		1
Firmicutes	*Bacillus*	38	24	*B. aryabhattai*	2	
				B. horneckiae		1
				B. litoralis		1
				B. marisflavi		1
				B. megaterium		1
				B. niacini		1
				B. oceanisediminis		2
				B. pumilus	34	
				B. thuringiensis	2	17
	Lysinibacillus		8	*L. fusiformis*		6
				L. parviboronicapiens		1
				L. varians		1
	Viridibacillus		9	*V. arenosi*		9
Proteobacteria	*Caulobacter*	1		*C. segnis*	1	
	Cedecea		1	*C. neteri*		1
	Enterobacter		4	*E. cancerogenus*		2
				E. cloacae		1
				E. ludwigii		1
	Ochrobactrum		1	*O. anthropi*		1
	Pseudomonas	24	3	*P. aeruginosa*	2	
				P. brassicacearum	5	
				P. fluorescens		1
				P. plecoglossicida		1
				P. stutzeri		1
				P. thivervalensis	17	
	Rheinheimera		1	*R. chironomi*		1
	Rhizobium	6		*A. tumefaciens*	6	
	Stenotrophomonas	8	1	*S. rhizophila*		1
				S. chelatiphaga	8	

Supplementary Table S2. Percentage distribution of plant growth promoting activities according to the bacterial genera isolated from the root system of *P. australis*. IAA = 3-indoleacetic acid production, ACC-d = ACC deaminase activity, Protease = protease production, EPS = exopolysaccharides production, SWIMMING =swimming lifestyle, SWARMING = swarming lifestyle.

Genus	n° strain	IAA	ACC-d	Protease	EPS	Swimming	Swarming
Arthrobacter	1	0	100	100	0	0	0
Microbacterium	4	50	100	25	25	0	0
Streptomyces	4	50	50	0	0	0	0
Flavobacterium	10	20	50	20	20	0	0
Fluviicola	1	0	0	0	0	100	100
Bacillus	12	83	58	75	8	17	33
Lysinibacillus	7	100	57	57	0	86	86
Viridibacillus	2	100	100	0	0	100	100
Caulobacter	1	100	100	0	0	0	0
Cedecea	1	100	100	0	0	0	0
Enterobacter	4	100	100	0	0	75	25
Ochrobactrum	1	100	100	0	0	0	0
Pseudomonas	6	100	83	50	50	17	0
Rheinheimera	1	100	100	100	0	0	0
Stenotrophomonas	3	100	33	100	33	0	0

Supplementary Table S3. Metal-tolerance phenotypes. The code of the isolates is simplified and includes only the fraction of origin (R = rhizosphere; E = endosphere) and the progressive number. The percentage in the last column is referred to the strains belonging to each detected phenotype. '+': refers to the strain tolerance to the metal at the indicated concentration.

		**Metal Tolerance**									
		**$ZnCl_2$ (mM)**			**$NiCl_2$ (mM)**			**$CdCl_2$ (mM)**			
**Phenotype**	**Code of the isolates**	**0.5**	**1**	**2**	**0.5**	**1**	**2**	**0.05**	**0.5**	**1**	**% of isolates**
**1**	R8, R25, R31, R32, R34, R67	+	+	+	+	+	+	+	+	+	12
**2**	R17, R20, R78	+	+	+	+	+		+	+	+	6
**3**	R3, R6, R50	+	+	+	+	+	+	+	+		6
**4**	R15	+	+		+	+	+	+	+		2
**5**	R22, R68	+	+	+	+	+		+	+		4
**6**	E2, E6, E8, E14, E16, E21, E28, E33, E73, R4, R28, R65, R71, R75	+	+	+	+	+	+	+			28
**7**	E13, R76, R79	+	+	+	+	+		+			6
**8**	R26, R69	+	+		+	+	+	+			4
**9**	R2	+	+		+	+		+			2
**10**	R16, R23, R38, R39, R47, R49, R52, R64	+	+	+	+	+	+				16
**11**	E15, R10, R40	+	+	+	+	+					6
**12**	R33	+	+		+	+	+				2
**13**	R7	+			+	+	+	+			2
**14**	E27	+	+		+	+					2
**15**	R77	+						+			2

Supplementary Table S4 (next page). Characterization of the bacterial collection.

In vitro plant growth promotion (PGP) tests: IAA = IAA production; ACC-d = ACC deaminase activity; Prot. = protease production. *In vivo* PGP test under hydroponic condition with *L. esculentum* plants: % germin. = germination percentage; root-l = root length; shoot-l = shoot length; 2ary roots = production of secondary roots; SVI = seedling vigor index; root dw = root dry weight. Ability to bind the root apparatus: Swimming = swimming lifestyle; Swarming = swarming lifestyle; EPS exopolysaccharides release. BPA tolerance. In vitro decolorization ability of RB5 = Reactive Black 5.

The + indicates that the bacterium is positive to the test. The stars indicate statistically significant differences (* $p < 0.05$, ** $p < 0.01$, *** $p < 0.001$). R indicates the resistance to the antibiotic.

Strain	Closest Described Relative	IAA	ACC-d	Prot.	Hydroponic Experiment						Swimming	Swarming	EPS	BPA	RB5
					% Germin.	Root-l	Shoot-l	2ary Roots	SVI	Root-dw					
CWMP-8E2	*Pseudomonas thivervalensis*	+	+	+									+	+	
CWMP-8E6	*Flavobacterium johnsoniae*	+	+	+									+	+	
CWMP-8E8	*Stenotrophomonas chelatiphaga*	+	+	+									+	+	
CWMP-8E13	*Bacillus thuringiensis*			+								+		+	
CWMP-8E14	*Flavobacterium gyeonganense*		+		*			**						+	
CWMP-8E15	*Bacillus aryabhattai*	+	+	+											
CWMP-8E16	*Flavobacterium gyeonganense*													+	
CWMP-8E21	*Pseudomonas thivervalensis*	+	+	+				**					+	+	
CWMP-8E27	*Bacillus aryabhattai*	+	+	+										+	
CWMP-8E28	*Pseudomonas brassicacearum*	+	+	+									+	+	
CWMP-8E33	*Bacillus pumilus*	+		+									+		
CWMP-8E42	*Caulobacter segnis*	+	+					**						+	
CWMP-8E73	*Stenotrophomonas chelatiphaga*	+		+											
CWMP-8R1	*Bacillus niacini*	+												+	
CWMP-8R2	*Microbacterium kitamiense*		+				**		*	*				+	1%
CWMP-8R3	*Bacillus thuringiensis*	+	+	+							+	+		+	24%
CWMP-8R4	*Flavobacterium oncorhynchi*		+							*				+	17%
CWMP-8R6	*Flavobacterium johnsoniae*		+							**				+	7%
CWMP-8R7	*Bacillus megaterium*	+	+	+	**	*	***		***	*				+	14%
CWMP-8R8	*Enterobacter cancerogenus*	+	+			*	***	**	**	*	+	+		+	32%
CWMP-8R9	*Streptomyces althioticus*						***							+	

CWMP-8R10	*Viridibacillus arenosi*	+	+		*	**	***		***	**	+	+		+	9%
CWMP-8R12	*Streptomyces acidiscabies*		+			***	***		***					+	
CWMP-8R15	*Bacillus horneckiae*		+	+			**		*	*	+	+			29%
CWMP-8R16	*Lysinibacillus fusiformis*	+	+				**				+	+		+	11%
CWMP-8R17	*Enterobacter cloacae*	+	+								+	+		+	37%
CWMP-8R19	*Bacillus oceanisediminis*	+	+	+			*	*						+	
CWMP-8R20	*Cedecea neteri*	+	+			*	*		*					+	10%
CWMP-8R22	*Enterobacter cancerogenus*	+	+											+	35%
CWMP-8R23	*Lysinibacillus fusiformis*	+		+		***	***	**	*		+	+		+	18%
CWMP-8R26	*Flavobacterium gyeonganense*	+	+											+	13%
CWMP-8R28	*Flavobacterium johnsoniae*				*	*								+	5%
CWMP-8R31	*Microbacterium kitamiense*		+				*							+	6%
CWMP-8R32	*Ochrobactrum anthrop*	+	+					*	*					+	27%
CWMP-8R33	*Arthrobacter ureafaciens*		+	+										+	50%
CWMP-8R38	*Lysinibacillus varians*	+	+	+										+	22%
CWMP-8R39	*Lysinibacillus parviboronicapiens*	+		+		**			*		+	+		+	19%
CWMP-8R40	*Viridibacillus arenosi*	+	+			**	***		**		+	+		+	7%
CWMP-8R47	*Fluviicola taffensis*						***							+	20%
CWMP-8R49	*Lysinibacillus fusiformis*	+	+	+			***		*		+	+		+	18%
CWMP-8R50	*Pseudomonas plecoglossicida*	+	+	+										+	12%
CWMP-8R52	*Streptomyces pluricolorescens*	+			*		***		***					+	
CWMP-8R57	*Rheinheimera chironomi*	+	+	+		***	***		***					+	
CWMP-8R64	*Lysinibacillus fusiformis*	+	+				***				+	+		+	74%
CWMP-8R65	*Flavobacterium johnsoniae*						***							+	22%
CWMP-8R68	*Bacillus marisflavi*	+		+		*	***		***			+		+	10%
CWMP-8R69	*Pseudomonas stutzeri*	+				*	***		**					+	30%
CWMP-8R72	*Bacillus litoralis*	+	+											+	
CWMP-8R76	*Flavobacterium gyeonganense*			+		***	***		***					+	16%
CWMP-8R77	*Bacillus oceanisediminis*	+				*	***		***					+	
CWMP-8R79	*Stenotrophomonas rhizophila*	+		+		**	***		**					+	32%
CWMP-8R80	*Streptomyces caeruleatus*	+	+				**		*					+	

Supplementary Table S5. Effect of bacterial consortium on *Juncus acutus* plants grown in soil. The stars indicate statistically significant differences in comparison to the non bacterized plants (* $0.01 \leq p < 0.05$).

Treatment	Root			Shoot			
	Length (cm)	Fresh Weight (g)	Dry Weight (g)	Length (cm)	Fresh Weight (g)	Dry Weight (g)	Chlorophyll (mg/g of leaf)
NC	22	0,78	0,24	35	3,30	0,85	0,23
MIX	23	1,37 *	0,32 *	36	4,45	1,10	0,36 *

NC: negative control

Supplementary Figure S1. Difference in root apparatus development of (a) bacterized and (b) non bacterized tomato seedlings.

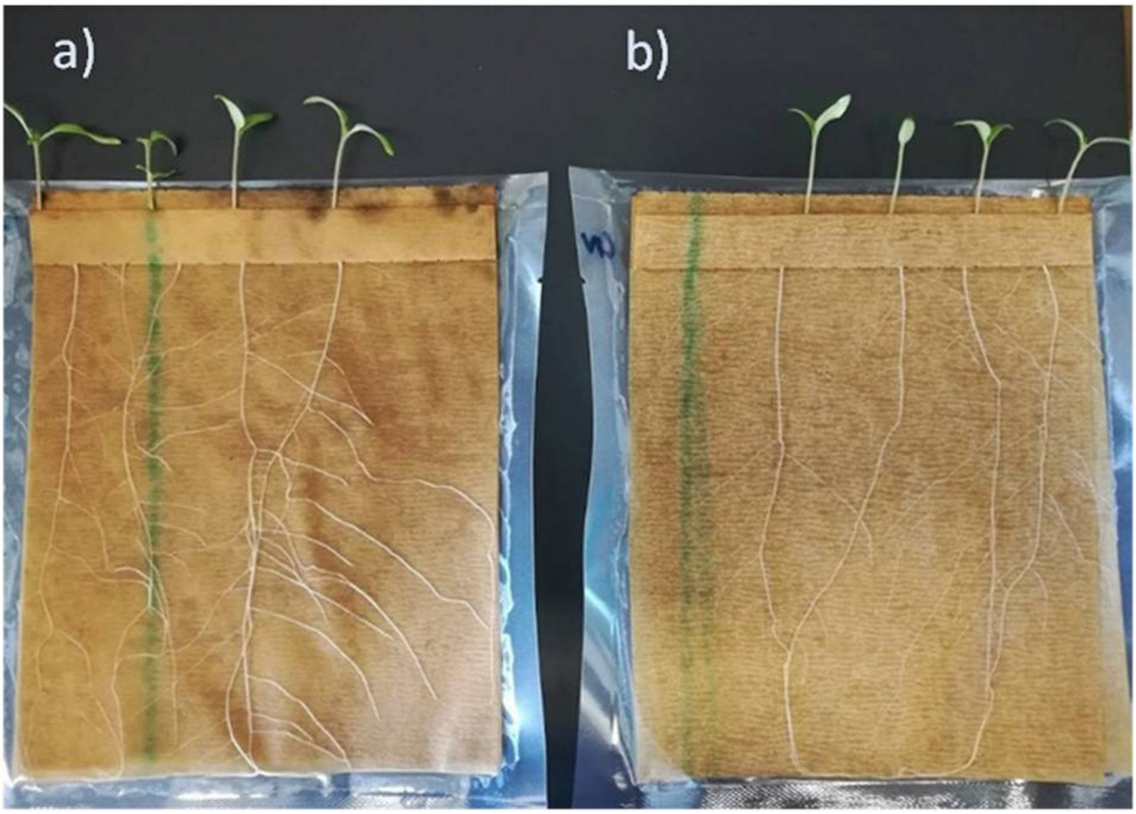

Supplement: Supplementary file 1 [file microorganisms-07-00384-s001.pdf]
